# Microproteinuria during *Opisthorchis viverrini* Infection: A Biomarker for Advanced Renal and Hepatobiliary Pathologies from Chronic Opisthorchiasis

**DOI:** 10.1371/journal.pntd.0002228

**Published:** 2013-05-23

**Authors:** Prasert Saichua, Paiboon Sithithaworn, Amar R. Jariwala, David J. Deimert, Jiraporn Sithithaworn, Banchob Sripa, Thewarach Laha, Eimorn Mairiang, Chawalit Pairojkul, Maria Victoria Periago, Narong Khuntikeo, Jason Mulvenna, Jeffrey M. Bethony

**Affiliations:** 1 Biomedical Sciences Program, Graduate School, Khon Kaen University, Khon Kaen, Thailand; 2 Department of Parasitology, Faculty of Medicine, Khon Kaen University, Khon Kaen, Thailand; 3 Liver Fluke and Cholangiocarcinoma Research Center, Faculty of Medicine, Khon Kaen University, Khon Kaen, Thailand; 4 Department of Microbiology, Immunology and Tropical Medicine and Center for the Neglected Diseases of Poverty, George Washington University, Washington, D.C., United States of America; 5 Department of Clinical Microscopy, Faculty of Associated Medical Sciences, Khon Kaen University, Khon Kaen, Thailand; 6 Department of Pathology, Faculty of Medicine, Khon Kaen University, Khon Kaen, Thailand; 7 Department of Radiology, Faculty of Medicine, Khon Kaen University, Khon Kaen, Thailand; 8 Insituto René Rachou, Laboratório de Imunologia Celular e Molecular, Belo Horizonte, Brazil; 9 Department of Surgery, Faculty of Medicine, Khon Kaen University, Khon Kaen, Thailand; 10 Infections and Cancer, Queensland Institute of Medical Research, Queensland, Australia; National Institute of Parasitic Diseases Chinese Center for Disease Control and Prevention, China

## Abstract

Approximately 680 million people are at risk of infection with *Opisthorchis viverrini* (OV) and *Clonorchis sinensis*, with an estimated 10 million infected with OV in Southeast Asia alone. While opisthorchiasis is associated with hepatobiliary pathologies, such as advanced periductal fibrosis (APF) and cholangiocarcinoma (CCA), animal models of OV infection show that immune-complex glomerulonephritis is an important renal pathology that develops simultaneously with hepatobiliary pathologies. A cardinal sign of immune-complex glomerulonephritis is the urinary excretion of immunoglobulin G (IgG) (microproteinuria). In community-based studies in OV endemic areas along the Chi River in northeastern Thailand, we observed that over half of the participants had urine IgG against a crude OV antigen extract (OV antigen). We also observed that elevated levels of urine IgG to OV antigen were not associated with the intensity of OV infection, but were likely the result of immune-complex glomerulonephritis as seen in animal models of OV infection. Moreover, we observed that urine IgG to OV antigen was excreted at concentrations 21 times higher in individuals with APF and 158 times higher in individuals with CCA than controls. We also observed that elevated urine IgG to OV antigen could identify APF+ and CCA+ individuals from non-cases. Finally, individuals with urine IgG to OV antigen had a greater risk of APF as determined by Odds Ratios (OR = 6.69; 95%CI: 2.87, 15.58) and a greater risk of CCA (OR = 71.13; 95%CI: 15.13, 334.0) than individuals with no detectable level of urine IgG to OV antigen. Herein, we show for the first time the extensive burden of renal pathology in OV endemic areas and that a urine biomarker could serve to estimate risk for both renal and hepatobiliary pathologies during OV infection, i.e., serve as a “*syndromic biomarker*” of the advanced pathologies from opisthorchiasis.

## Author Summary

Approximately 680 million people risk infection with food-borne trematodes, including *Opisthorchis viverrini* (OV). Animal models show that significant kidney pathology results from OV infection as detected by antibodies in urine (microproteinuria). However, kidney pathology in humans infected with OV is often overlooked because it develops alongside more severe pathologies such as bile duct fibrosis and bile duct cancer. In Northeastern Thailand, the researchers observed that OV infected individuals had elevated levels of urine IgG against OV antigen that was not associated with the level of OV infection. The researchers observed that urine IgG to OV antigen was associated with bile duct fibrosis and bile duct cancer. Moreover, individuals with urine IgG to OV antigen also had elevated risk of bile duct fibrosis and bile duct cancer than individuals with no urine IgG to OV antigen. For the first time, OV infection has been shown to result in significant kidney disease in humans, which is also strongly associated with bile duct pathology. A urine-based assay that could indicate both renal and bile duct pathology from OV infection would be of profound benefit in Southeast Asia, especially in the resource-limited settings of the Mekong Basin region countries of Thailand, Laos and Cambodia.

## Introduction

Foodborne trematodiases represent an important group of communicable diseases, and some of the most clinically significant neglected tropical diseases (NTDs) affecting East Asia. Approximately 680 million people are at risk of infection with the human liver flukes *Opisthorchis viverrini* and *Clonorchis sinensis* [1]. In Southeast Asia alone, up to 67 million people are at risk of infection with *O. viverrini* (OV), with 10 million people estimated to be infected with this pathogen in the Mekong Basin Subregion of Thailand and Lao PDR [2], [3]. Humans become infected with OV by consuming raw or undercooked fish that contain the infective metacercarial stage (for review see [4]). Although the infection can be eliminated by the anthelminthic praziquantel, environmental and cultural factors of the Mekong Basin region strongly favor re-infection [4]. Despite mass drug administration (MDA) efforts in the northeast region of Thailand (Isaan), the prevalence of OV remains intransigently high [5], [6].

Our community-based ultrasound studies in *O. viverrini* endemic areas along the Chi River Basin in Khon Kaen, Thailand have revealed that significant morbidity occurs early during the course of chronic OV infection, including advanced hepatobiliary pathologies such as advanced bile duct (periductal) fibrosis (APF) and bile duct cancer (cholangiocarcinoma or CCA) [7], [8]. As individuals do not become symptomatic until the late stages of these diseases, early detection remains an important public health objective [4], [6]. Although renal disease is not usually considered among the more critical pathologies of chronic opisthorchiasis, as with many other parasitic infections (e.g. *Plasmodium spp, Schistosoma spp, Filarioidea*) [9], glomerulopathy has been reported in laboratory animal models of OV infection [10], [11]. More specifically, early during experimental OV infection (8 weeks), hamsters develop a “mesangioproliferative glomerulonephritis”, characterized by the deposition of immune complexes (ICs) consisting of immunoglobulin (Ig) G, complement component 3 (C3), and OV tegumental antigen [10]. After 12 weeks, the infected hamsters show a complete obsolesce of the glomeruli characterized by deposition of amyloid (AA protein), tubular atrophy, interstitial inflammation, and tubular fibrosis, all of which are co-incident with APF and CCA [10]. It is interesting to note that a deterioration in renal function has been reported in humans with obstructive jaundice due to OV-associated CCA in endemic areas of Thailand [12], although this is likely a manifestation of ‘hepatorenal syndrome’ (HRS), a common end stage complication of chronic hepatic diseases, such as liver cirrhosis and liver cancer [13].

Previous studies have attempted to show a correlation between the intensity of OV infection and levels of urine IgG to various crude OV antigen extracts [14]–[16]. Although urine can contain small quantities of ‘intact’ immunoglobulin as well as light and heavy chain fragments of immunoglobulin, the restrictive pore radius of the renal glomerular filter in a healthy human kidney would not filter macromolecules the size of intact IgG (for review see [17]). As such, the frequent observation of elevated levels of urine IgG to OV antigen in areas of high OV transmission [14]–[16] most likely reflects structural damage from immune complex deposition in the glomeruli as observed in the hamster model of OV infection [10], [11]. In the current manuscript, we investigated the presence of urine IgG to a crude adult OV antigen extract (OV antigen) in residents from OV endemic areas along the Chi River Basin, in Khon Kaen Thailand. Our hypothesis is that if levels of urine IgG to OV antigen are elevated in individuals with renal and hepatobiliary pathologies, then this non-invasive and easily assayed biomarker could serve as a single marker for both pathologies, i.e., as a “*syndromic biomarker*” of advanced pathologies from chronic opisthorchiasis.

## Materials and Methods

### Study sample and study design

This study uses baseline data from the Khon Kaen Cancer Cohort (KKCC), which was conducted in seven (7) villages with high OV transmission along the Chi River Basin in Khon Kaen Thailand. A detailed description of the KKCC and the methods used to assemble this cohort can be found in several manuscripts [7], [8], [18]. The dataset from the KKCC included 296 individuals divided into three clinical groups described below and shown in Table 1. In brief, 148 males and 148 females were enrolled in the KKCC. Of the males and females in this dataset, 256 (86.4%) were infected with OV as determined by microscopic fecal examination. Participants in the KKCC were classified into groups based on abdominal ultrasound (US) examination and microscopic fecal examination for OV infection. Group 1 consisted of 40 individuals considered “Endemic Normals” (EN), who were age, sex and ‘nearest-eligible-neighbor’ matched with cases (Group 3) and were OV negative (OV−) and APF negative (APF−) as determined by abdominal US. Group 2 consisted of 139 individuals considered “controls”, who were age, sex and ‘nearest-eligible-neighbor’ matched to cases (Group 3) and were OV positive (OV+) and APF negative (APF−). Group 3 consisted of 117 individuals considered “cases“ who were APF positive (APF+). Group 4 was not part of the KKCC and consisted of 98 individuals with histologically proven opisthorchiasis-associated CCA whose serum and urine samples were obtained from the biological specimen repository of the Liver Fluke and Cholangiocarcinoma Research Center, Khon Kaen University, Thailand. Individuals positive for infection with OV were referred to the local public health outpost for treatment with praziquantel.

**Table 1:** Characteristics of the study participants in the Khon Kaen Cancer Cohort (KKCC) and CCA cases.

	Group 1	Group 2	Group 3	Group 4	
Characteristic	Endemic Normal*	OV+ APF−†	APF+	CCA+‡	Total
N =	40	139	117	98	394
Sex					
Male	13 (32%)	76 (54.7%)	59 (50.4%)	59 (60%)	207
Female	27 (68%)	63 (45.3%)	58 (49.6%)	39 (40%)	187
Age					
Mean ± SD	49±7.55	47±8.32	47±10.17	59±9.52	
Intervals					
≤30	0 (0%)	2 (1%)	5 (4%)	0 (0.0%)	7
31–40	6 (15%)	30 (22%)	28 (24%)	4 (4%)	68
41–50	14 (35%)	63 (45%)	32 (27%)	13 (13%)	122
>50	20 (50%)	44 (32%)	52 (44%)	81 (83%	197
OV infection					
Egg/gram feces					
0	40 (100%)	–	–	–	40
1–499	–	123 (88%)	97 (83.%)	–	220
≥500	–	16 (12%)	20 (17%)	–	36

* Endemic Normal refers to individuals who are “negative” for OV infection and for APF;

† Advanced Periductal Fibrosis as determined by abdominal ultrasound.

‡ Cholangiocarcinoma.

Study participant recruited from *O. viverrini* endemic areas along the Chi River Basin in Khon Kaen, Thailand from 2010 to 2012, as part of the Khon Kaen Cancer Cohort (KKCC). This includes individuals with confirmed OV-associated cholangiocarcinoma (CCA) from the biological specimen repository of the Liver fluke and Cholangiocarcinoma Research Center, Khon Kaen University, Thailand.

### Ethics statement

All subjects in Groups 1–3 provided written informed consent using forms approved by the Ethics Committee of Khon Kaen University School of Medicine, Khon Kaen, Thailand (reference number HE480528) and the Institutional Review Board of the George Washington University School of Medicine, Washington, D.C (GWUMC IRB# 020864). The serum and urine from Group 4 was obtained from the biological specimen repository of the Liver Fluke and Cholangiocarcinoma Research Center, Khon Kaen University, Thailand using a protocol approved by the Ethical Committee on Human Research, Faculty of Medicine, Khon Kaen University, Thailand (reference Nos. HE450525 and HE531061).

### Clinical assessment and specimen acquisition

Assessment of hepatobiliary status was done by abdominal US with positive findings scored as APF+ or APF− as previously described [7], [8]. Two fecal samples were collected on consecutive days from each participant in Groups 1–3; fecal samples were not available for Group 4 patients (CCA cases). OV infection was determined and quantified (eggs per gram of feces or epg) by microscopic fecal examination using the formalin-ethyl acetate concentration technique (FECT) as described by Elkins et al [19] on two consecutive days of fecal samples. In addition, the following samples were also collected from Groups 1–3: thirty (30) milliliters (ml) of venous blood collected into siliconized tubes after overnight fasting and first morning mid-stream urine samples collected into sterile containers. Venous blood samples were allowed to clot at room temperature for 30 minutes after collection, centrifuged, and the serum removed and aliquoted for storage stored at −20°C in a temperature-monitored freezer. Urine samples were centrifuged and the supernatant aliquoted and stored at −20°C in a temperature-monitored freezer. In the case of CCA patients (Group 4), serum or urine specimens were obtained by simple random sampling from the collection of biological specimens in the repository of the Liver fluke and Cholangiocarcinoma Research Center, Khon Kaen University, Thailand.

### Point of care testing for proteinuria

Individual subjects were asked to provide morning urine into clean polypropylene containers that were kept on ice during transportation to the laboratory. Unprocessed urine samples were screened for protein by urine strip (ARKRAY's AUTION Sticks, Japan) and then analyzed using an Automated Urine Chemistry Analyzer (AUTION MAX AX-4280, Arkray, USA).

### Preparation of crude adult *O. viverrini* antigen extract

Adult *O. viverrini* worms from experimentally infected hamsters were washed three times with sterile phosphate buffered saline (PBS pH 7.2) containing 0.149 M sodium chloride (Fisher Scientific, NJ), 8.29 mM disodium hydrogen phosphate (Acros Organics, NJ) and 18 mM sodium dihydrogen phosphate monohydrate (Fisher Scientific, NJ) in deionized (DI) water. A 100× Protease Inhibitor Cocktail (Calbiochem, CA) was added to the worms in PBS, which were then homogenized using a tissue grinder on ice. The worm pellet was homogenized by ultrasonic disintegrator (MISONIC sonicator 3000, US) and then centrifuged at 4°C, 14000 rpm for 30 min. The BCA™ Protein Assay kit (PIERCE, IL) was used to determine the protein yield of the crude somatic *O. viverrini* adult antigen extract. The supernatant was collected and stored at −80°C until used.

### Development of a qualified indirect enzyme-linked immunosorbent assay (ELISA) for quantification of antibodies to a crude OV adult antigen extract

Flat-bottom 96-well microtiter plates (Maxisorb, NUNC, DN) were coated with 1 µg/ml of crude somatic *O. viverrini* adult antigen in PBS buffer (pH 7.2), which was then covered with sealing film and incubated overnight at 4°C in the dark. On the next day, the plates were washed 5 times with a buffer containing 0.05% Tween20 in PBS (pH 7.2) using an automated plate washer (Thermoelectron, MA). After washing, 250 µl of a blocking buffer containing 5% BSA (Fitzgerald, MA) in PBS and 0.5% Tween-20 (Fisher, NJ) was added to all wells, and the plates incubated at room temperature (RT) for 1 hour. Serum samples were diluted in a buffer which contained 5% BSA (Fitzgerald, MA) in PBS and 0.5% Tween-20 (Fisher, NJ), and added to wells (100 µL/well) in duplicates and incubated overnight at 4°C. Undiluted urine supernatants were added to wells (100 µL/well) in duplicate and incubated overnight at 4°C. The plates were then washed 5 times with a buffer of PBS and 0.5% Tween-20, and a horseradish peroxidase (HRP)-conjugated secondary antibody was added to all wells and incubated for 2 hours at RT. HRP-goat anti human IgG (Zymed, CA) was used to detect IgG in serum and urine. HRP-mouse anti-human IgG1 (Southern Biotech, AL), and an HRP-mouse anti-human IgG4 (Zymed, CA) were used to detect IgG1 and IgG4 in serum, respectively. After incubation and washing, a substrate solution, which consisted of Ortho phenylenediamine (Sigma, MO), 53 mM citric acid anhydrous (Fisher, NJ), 102 mM dibasic sodium phosphate dodecahydrate (Acros Organics, NJ) and 30% w/w hydrogen peroxide (Fisher, NJ) in DI water was added to the wells and incubated at RT in the dark for 30 min. The reaction was stopped by the addition of 2N sulfuric acid (BDH, PA) and the plates were read using a plate reader (SpectraMax 340PC^384^ system) at 492 nm.

### Development of an indirect ELISA to measure IgG to OV-antigen in urine and serum

Following the method of Quinn and colleagues [20]–[22], we developed a diagnostic assay using an indirect ELISA that incorporates “homologous interpolation” to determine the concentration of an analyte (e.g., anti-OV IgG) in either diluted serum or undiluted urine supernatant samples by interpolation of test serum or urine supernatant OD at 492 nm onto a Standard Calibration Curve (SCC) run on each microtitre plate. Briefly, a Standard Reference Sera (SRS) and urine Standard Reference Solution (SRS) were made by pooling of sera or urine supernatants with known high levels of IgG and its subclasses against Ov antigen from individuals who were *O. viverrini* egg positive (see references for details of this method [20]–[22]). Each serum SRS and the urine SRS are serially diluted on each microtiter plate in two-fold steps using a dilution buffer (5% Bovine Serum Albumin in PBS and 0.5% Tween-20 at a pH of 7.2). The ODs of each dilution point are then used to generate the SCC by 4-PL regression modeling (SOFTmax PRO version 5.4 software) [23], [24]. To generate the SCC, Arbitrary Units (AU) of antibody are assigned to the Standard Calibration Curves as shown in Table S1. The 4-PL function is used to model the characteristic curve for the SRS. As shown by Quinn et al [22], the SRS in serial dilution should exhibit a sigmoidal shape when plotted on an OD-log10 dilution scale. The 4-PL function fits these data with a high degree of accuracy and extends the range of the assay, thus providing a more precise measurement of antibody concentration for patient sera [20]–[22]. Furthermore, 8 wells per ELISA plate were assigned as internal controls, consisting of two blanks (no sample with/without secondary antibody), a positive serum and urine control, and negative serum and urine control. The ELISA was qualified for accuracy and precision as previously described [22].

### Reliable Detection Limit (RDL) of the indirect ELISA

Figure S1 shows a graphic representation of the methods used to derive the RDL, which was used as the threshold above which a serum or urine sample was considered positive for antibodies against OV antigen. Figure S1 Panels A, C, E and G show the mean of the combined SCCs for each antibody-antigen pair (e.g., serum IgG1 to OV antigen) and their 95% CI intervals. Figure S1 Panels B, D, F and H show the derivation of the RDL. In a manner similar to Quinn et al. [22], the RDL was derived from the level of AUs for each anti-OV-antigen antibody corresponding to the interpolated intersection of the upper 95% CI asymptote with the lower-95% CI of the standards data as shown Figure S1 Panels B, D, F, and H. A human serum or urine supernatant sample with Arbitrary Antibody units above the RDL was defined as “reactive” (positive) and those with Arbitrary Antibody units below the RDL as “nonreactive” (negative) as shown in Table 2.

**Table 2:** Serum and urine antibodies to an *Opisthorchis viverrini* crude antigen extract by clinical group.

		Serum							Urine	
		IgG		IgG1		IgG4			IgG	
RDL‡ (AU) =		3.9		2.6		5.0			2.7	
		Pos	Neg	Pos	Neg	Pos	Neg		Pos	Neg
	N	N (%)	N (%)	N (%)	N (%)	N (%)	N (%)	N	N (%)	N (%)
EN†	40	**40** (100)	**0** (0)	**37** (95)	**2** (5)	**6** (35)	**11** (65)	15	**5** (33)	**10** (67)
OV+APF−	139	**139** (100)	**0** (0)	**139** (100)	**0** (0)	**52** (51)	**50** (49)	79	**30** (38)	**49** (62)
APF+	117	**117** (100)	**0** (0)	**117** (100)	**0** (0)	**59** (51)	**57** (49)	103	**64** (62)	**39** (38)
CCA+	98	**98** (100)	**0** (0)	**94** (96)	**4** (4)	**11** (37)	**19** (63)	8	**8** (100)	**0** (0)

‡ RDL or Reliable Detection Limit as shown in Supporting Figure 1 Panels B, D, F and H.

† The term EN refers to OV− and APF− individuals resident in OV endemic areas along the Chi River Basin in Khon Kaen, Thailand.

**Figure 1:**
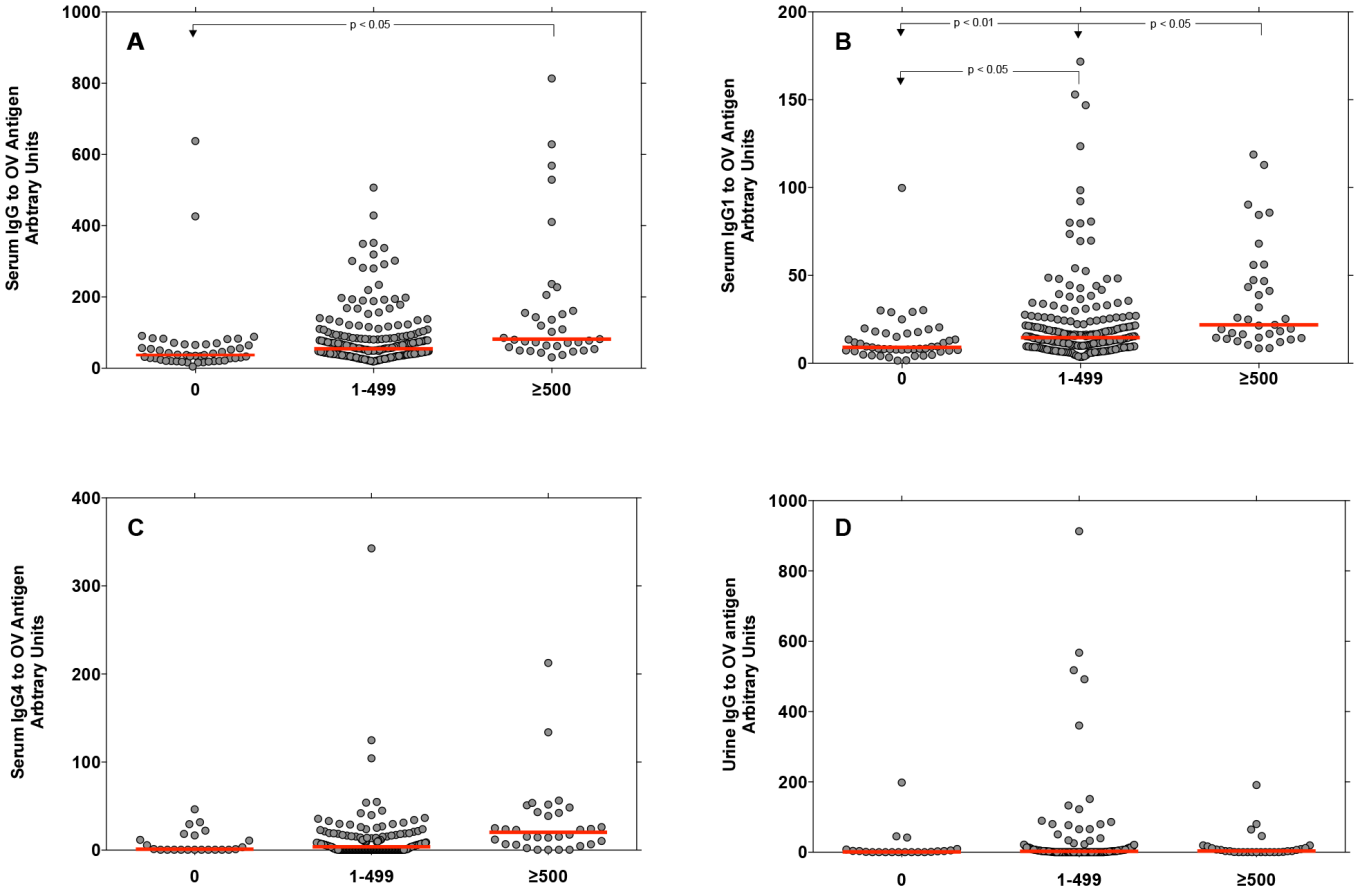
The relationship between *Opisthorchis viverrini* infection and serum and urine antibodies to OV antigen. The status of *O. viverrini* infection was determined by microscopic fecal examination. Individuals were categorized as to the intensity of OV infection by the geometric mean of the eggs per gram of feces as follows: “negative” or “0” (no eggs detected in feces), “lightly infected (1–499 eggs per gram of feces), or “medium-to heavily“ infected (≥500 eggs per gram of feces). In the case of CCA patients, the serum and urine specimens were obtained from histologically proven cases of opisthorchiasis-associated cholangiocarcinoma from the biological specimen repository of the Liver fluke and Cholangiocarcinoma Research Center, Khon Kaen University, Thailand. The levels of the following antibodies were determined to a crude adult OV antigen extract by indirect ELISA: serum IgG (**Panel A**), serum IgG1 (**Panel B**), serum IgG4 (**Panel C**) and urine IgG (**Panel D**). The Ab levels of each infection group was estimated by the mean shown as the red horizontal line in each group and tested using ANOVA followed by Pairwise Testing of each group with a Bonferroni correction for multiple testing.

Positivity is determined by serum or urine samples having antibodies over the Reliable Limit of Detection (see Figure 1 Panels B, D, F, and H). Advanced periductal fibrosis (APF) was determined by the “Gold Standard of abdominal ultrasound, and OV positivity by the “Gold Standard” of microscopic fecal exam. The CCA cases were from (CCA) from the biological specimen repository of the Liver fluke and Cholangiocarcinoma Research Center, Khon Kaen University, Thailand.

### Indirect ELISA performance characteristics

Again following Quinn et al., [22], we defined ELISA assay “accuracy” as the exactness of the assay to measure a known, true value of urine anti-OV IgG and to measure it repeatedly and expressed assay accuracy as the percent error between the assay-determined values and the assigned value for that serum. A percent error of ≤20% was considered the acceptable level of accuracy for the ELISAs presented herein [22], [25]. We also defined assay “precision” according to Quinn et al [22], [25] as the measure of the degree of repeatability of an assay under normal operating conditions, and expressed assay precision as the coefficient of variation (CV) of the concentrations calculated for the SCC dilutions within a single assay plate (intra-assay precision) and between different assay plates (inter-assay precision) determined over time and controlling for different operators. Acceptable levels of intra-assay and inter-assay precision are 10% and 20%, respectively [22], [25]. The “goodness of fit” of each SCC was used to determine how closely each SRS fit the 4-PL model. Goodness of fit was expressed as the regression coefficient (*R^2^*) of the SCC. An *R^2^* value that approached unity (1.00) was indicative of a good fit for the data to the curve [22], [25] and these are shown for each SCC in Figure S1 Panels A, C, E and G.

### Clinical data analysis

Data distributions were assessed for normality. For normally-distributed data, differences between AUs of antibody to OV antigen were compared between different matrices (urine or sera) by clinical groups or by different matrices using the intensity of OV infection by Student's t-test. Non-parametric data were compared using the Mann-Whitney U-test. One-way ANOVA (normally distributed data) or a Kruskal-Wallis tests (non-normally distributed data) were used to determine statistically significant associations among the aforementioned groups followed by a Bonferonni corrected pairwise comparison when comparing pairs in each group. All statistical analyses were performed using SAS 9.2. Results were considered significant when the p-value was less than <0.05.

Sensitivity was calculated as the number of individuals with serum or urine IgG to OV antigen above the cut-off set for each assay as determine by Receiver Operator Characteristic (ROC) curves obtained with ROCKIT1.1 software. Specificity was calculated as the number of individuals with serum or urine IgG to OV antigen below the “cut-off” set for each assay divided by the total number of control individuals (EN or APF−). As we propose these assays as screening tests, we selected the cut-offs to achieve the highest sensitivity without losing specificity: i.e., the best trade off between high sensitivity and modest specificity. The area under the curve (AUC) is a measure of the ROC's validity. The 45-degree line in each ROC curve analysis subsumed an area equal to 0.50 and is equivalent to using a coin toss procedure to classify participants. To calculate the positive predictive values (PPV) for each assay, we used a 50% prevalence of infection OV as determined by microscopic fecal exam in the age range of 20 to 60 years from our previous studies of the KKCC [7], [8], [18]. The following formula was used to estimate the Positive Predictive Value with prevalence set at 0.50 for each:
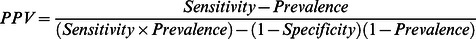



## Results

### Characteristics of the study sample

Table 1 shows the age of the study participants by clinical group. Of the two hundred and ninety-six (296) individuals who provided fecal specimens for examination by FECT (Groups 1, 2 and 3), 256 (86.4%) were confirmed to be infected with OV by microscopic fecal exam. One hundred and seventeen individuals (n = 117) were assigned the status of APF+ and assigned the status of case. Additionally, 139 OV+ and APF− individuals were assigned the status of age, sex and “nearest-eligible-neighbor” matched controls (Group 2). Note that the larger sample size of the controls is due to the fact that 22 of the cases were matched with two controls for greater sample size. Forty (n = 40) OV− and APF− individuals were assigned the status of Endemic Normals. Finally, 98 serum samples were obtained by simple random sampling from the biological specimen bank of the Liver Fluke and Cholangiocarcinoma Research Center, Khon Kaen University, Thailand; only 8 of these individuals has urine samples. Fecal specimens were not available for the CCA patients.

### Performance characteristics of the anti-OV IgG ELISA

Figure S2 shows the parallelism of each SCC by plotting linearized versions of each SCC for serum IgG (*P* = 0.225) (Figure S2 Panel A), serum IgG1 (*P* = 00.240) (Figure S2 Panel B), serum IgG4 (*P* = 0.136) (Figure S2 Panel C), and urine IgG (*P* = 0.402) ( Panel D). The regression slopes of the fitted SCCs were not significantly different as determined by ANOVA (p>0.05), indicating parallelism among each group of SCCs [24].

### Serum IgG and IgG1 against OV antigen were detected in nearly all individuals (100% and 98%, respectively) including individuals in the Endemic Normal (EN) group

Using the RDL shown in Figure S1 Panel B as the detection threshold, Table 2 shows that serum IgG against OV antigen was detected in all individuals (100%; n = 394) in the study. Similarly, Table 2 also shows that using the RDL shown in Figure S1 Panel D as the detection threshold for anti-IgG1 OV antigen, nearly all (98%; n = 387) of individuals in the study had detectable levels of this serum antibody to OV antigen, again, regardless of OV or clinical status. Finally, using the RDL shown in Figure S1 Panel F as the detection threshold, Table 2 shows that a third to half of the individuals in each clinical group had detectable levels of IgG4 to OV antigen.

### Only half of APF+ individuals had detectable levels of urine IgG to OV antigen

Using the RDL as the detection threshold (Figure S1 Panel H), Table 2 shows that over 60% of APF+ individuals had detectable levels of IgG to OV antigen in their urine. A lower proportion (38%) of individuals who were OV+ and APF− (matched controls) had detectable levels of urine IgG to OV antigen. All eight (n = 8) of the CCA patients who had urine samples had detectable levels of IgG to OV in their urine.

### Levels of serum IgG and IgG1 were significantly elevated in individuals with medium to heavy OV infection (≥500 eggs per gram of feces, epg) compared to EN group and to individuals with lighter levels of OV infection (1–499 epg)

Levels of serum IgG (Figure 1 Panel A) and serum IgG1 (Figure 1 Panel B) to OV antigen were significantly higher (P<0.001, for both) in individuals with both lighter (1–499 epg) or heavier (≥500 epg) OV infections compared to EN individuals (no eggs in feces). In addition, individuals with heavier OV infection had higher levels of serum IgG1 to OV antigen than individuals with lighter OV infection (Figure 1 Panel B). Levels of urine IgG were not significantly elevated in any of the infection groups (Figure 1 Panel D).

### Urine IgG to OV antigen was significantly elevated in individuals with advanced periductal fibrosis (APF+) or with histologically proven CCA compared to EN and APF− controls

**Figure 2:**
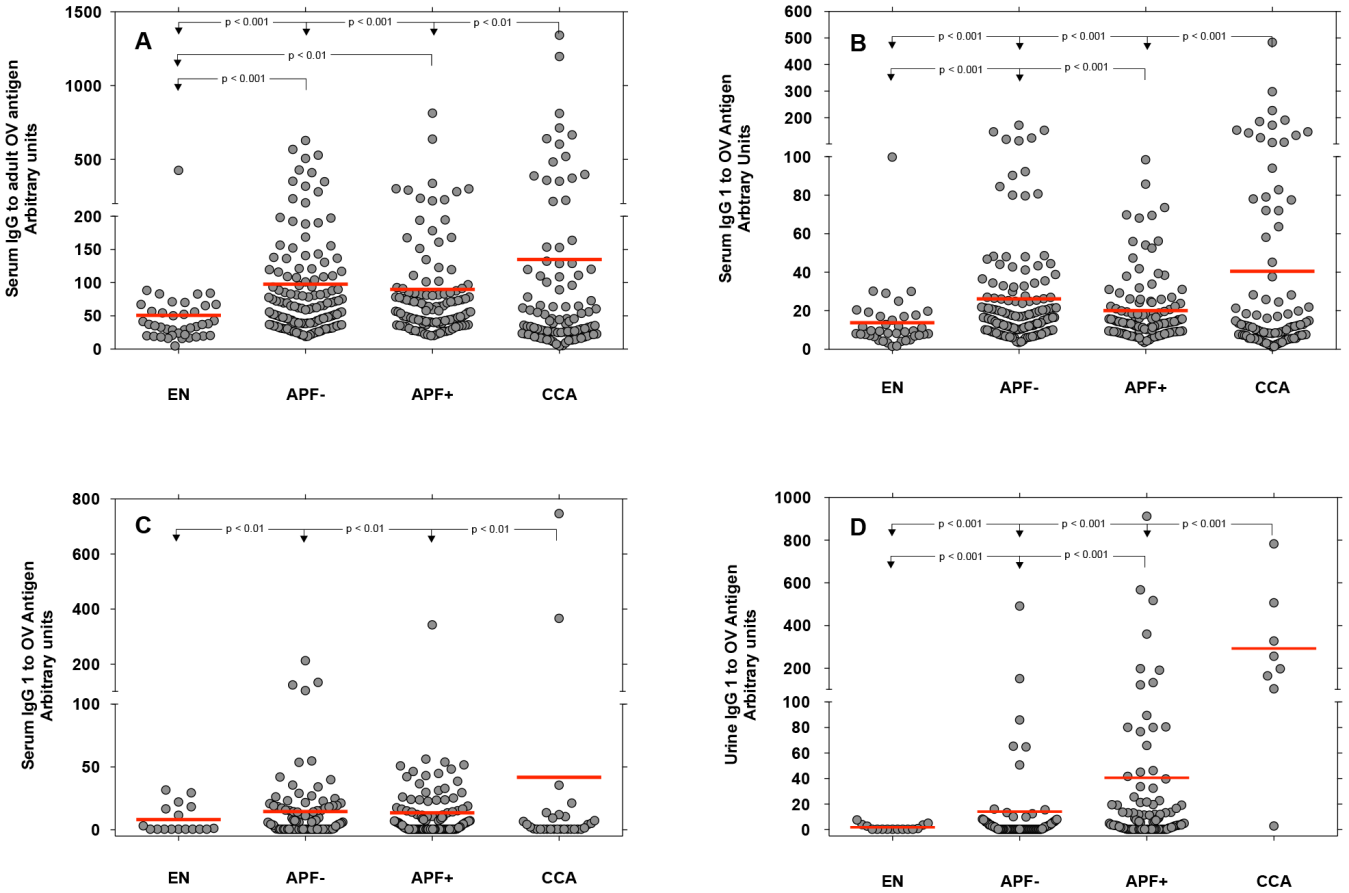
The relationship between hepatobiliary pathologies and levels of serum and urine antibodies to OV antigen. Individuals who were positive for *O. viverrini* but negative for APF by abdominal US were defined as “controls” and matched with cases by age (by ten year bands), sex, and nearest eligible neighbor method. The levels of the following antibodies (Ab) were determined to a crude adult OV antigen extract by indirect ELISA: serum IgG (**Panel A**), serum IgG1 (**Panel B**), serum IgG4 (**Panel C**) and urine IgG (**Panel D**). The Ab level of each infection group was estimated by the mean shown as the red horizontal line in each group and tested using analysis of variance (ANOVA) followed by pairwise testing of each group with Bonferroni correction for multiple testing.

Figure 2 Panel D shows that urine levels of IgG to OV antigen were significantly higher (P<0.001) in APF+ individuals than individuals in the EN or APF− groups: i.e., on average, 21 times higher in APF+ individuals than EN individuals and 7 times higher in APF+ individuals than APF− individuals. Similarly, Figure 2 Panel D shows that urine levels of IgG to OV antigen were significantly higher (P<0.001) in CCA+ individuals than individuals in the EN, APF−, and APF+ groups. On average, urine levels of IgG to OV antigen were 158 times higher in CCA+ individuals than EN individuals; 21 times higher in CCA+ individuals than APF− individuals; and 7 times higher in CCA+ individuals than APF+ individuals.

### No association was observed between serum levels of IgG to OV antigen and urine levels of IgG to OV-antigen

Figure S3 Panel A shows that there is no association between levels of serum IgG to OV antigen and urine levels of IgG to OV antigen in the same individuals. This was found for all infection and clinical categories. Figure S3 Panel B shows that few of OV+ individuals in either group (APF− or APF+) had proteinuria as determined by point-of-care testing using a strip-based urine reagent device, with a positive urine dipstick test for protein defined by a color change of “+” or greater that equates to at least 30 mg/L of protein.

### Elevated levels IgG1 to OV-antigen had the best area under the curve (AUC) as well as the highest positive predictive value (PPV) for OV infection

Table 3 shows the area under the curve (AUC) for ROC curve analyses with PPVs determined using 50% prevalence: e.g., serum IgG against OV antigen had an AUC of 0.68 and a PPV of 0.60 using the cutoff of 35.66 AUs resulting from ROC curve analysis of the highest possible sensitivity without a decrease in the specificity of the assay. Using these cutoffs, we estimated crude and adjusted Odds Ratios (95%CIs) for the risk of OV-positivity. The ROC curves for serum IgG1 against OV-antigen had the best AUC with 0.68 using an antibody cutoff of 9.21 AUs. Using this cutoff resulted in a PPV of 0.61, as well as an adjusted OR of 2.51 (95%CI 1.26, 5.00) for the risk of OV positivity. Despite a poor AUC and a weak PPV, urine IgG indicated significant risk for OV infection with a crude OR of 7.60 (95%CI 3.56, 16.20) and an adjusted OR of 7.68 (95%CI 3.58, 16.50).

**Table 3:** Clinical epidemiology of serum and urine IgG to OV antigen for the detection of OV infection.

						Odds Ratio (95%CI Lower, Upper)	
Assay	AUC†	Cut Off (AUs*)	Sensitivity (95%CI)	Specificity (95%CI)	PPV**	Crude	Adjusted^a^
Serum IgG	**0.68**	>35.66	**0.80** (0.75, 0.85)	**0.46** (0.31, 0.61)	**0.60**	**2.50** (1.26, 4.96)	**2.50** (1.25, 4.98)
Serum IgG1	**0.68**	>9.21	**0.80** (0.75, 0.85)	**0.49** (0.34, 0.64)	**0.61**	**2.57** (1.29, 5.10)	**2.51** (1.26, 5.00)
Serum IgG4	**0.51**	>8.37	**0.61** (0.52, 0.69)	**0.33** (0.10, 0.65)	**0.48**	**1.16** (0.58, 2.31)	**1.03** (0.51, 2.06)
Urine IgG	**0.56**	>3.74	**0.70** (0.61, 0.79)	**0.33** (0.10, 0.651)	**0.51**	**7.60** (3.56, 16.20)	**7.68** (3.58, 16.50)

† Area Under the Curve;

* Arbitrary Units of antibody;

** Positive predictive value;

a Adjusted for age and sex.

The detection of OV infection was determined by microscopic fecal exam. The positive predictive value (PPV) was estimated using (50%) prevalence from field studies in [7], [8], [18]. Odds Ratios and their 95% Confidence Intervals were based on the “cut-offs” obtained from Receiver Operator Characteristic (ROC) curve analyses Odds Ratios were adjusted for age and sex. Odds Ratios calculated against individuals with no detectable levels of antibody in urine.

### Elevated levels IgG1 to OV antigen also had the best AUC as well as the highest PPV for heavy OV infection

Table 4 shows that elevated levels of serum IgG1 to OV antigen had the best combination of sensitivity and specificity for the prediction of heavy OV infection (>500 epg) compared to individuals negative for OV. In addition, elevated levels of serum IgG and IgG1 to OV antigen showed excellent capacity to indicate risk of high OV infection as determined by significant crude and adjusted ORs (Table 4). Though serum IgG4 to OV antigen and urine IgG to OV antigen showed moderate discriminatory capacity for OV infection, Table 4 shows that elevated levels of urine IgG to OV antigen could still predict risk of OV infection as shown in an adjusted OR of 7.88 (95%CI: 2.64, 23.51).

**Table 4:** Clinical epidemiology of serum and urine IgG to OV antigen to detect levels of OV infection.

						Odds Ratio (95%CI Lower, Upper)	
Assay	AUC†	Cut Off (AUs*)	Sensitivity (95%CI)	Specificity 95%CI)	PPV**	Crude	Adjusted^a^
Serum IgG	**0.82**	>46.41	**0.91** (0.77, 0.98)	**0.59** (0.43, 0.73)	**0.69**	**7.90** (2.80, 22.33)	**9.62** (3.21, 28.79)
Serum IgG1	**0.83**	>12.04	**0.91** (0.77, 0.98)	**0.62** (0.47, 0.76)	**0.71**	**9.20** (3.19, 26.52)	**12.72** (4.11, 39.41)
Serum IgG4	**0.68**	>11.24	**0.79** (0.59, 0.92)	**0.42** (0.15, 0.72)	**0.57**	**4.82** (1.79, 12.95)	**4.23** (1.54, 11.85)
Urine IgG	**0.58**	>5.78	**0.68** (0.43, 0.87)	**0.58** (0.28, 0.85)	**0.62**	**6.09** (2.19, 16.90)	**7.88** (2.64, 23.51)

† Area Under the Curve;

* Arbitrary Units of antibody;

** Positive predictive value;

a Adjusted for age and sex.

The sensitivity and specificity of serum IgG, IgG1, and IgG4 and urine IgG to OV antigen extract for “medium-heavy” intensity of infection defined as ≥500 OV eggs per gram of feces by microscopic fecal exam. The positive predictive value (PPV) was estimated using (50%) prevalence from field studies in [7], [8], [18]. Odds Ratios and 95% Confidence Intervals were based on the “cut-off” points as obtained by Receiver Operator Characteristic (ROC) curve analysis. Odds Ratios were adjusted for age and sex. Odds Ratios calculated against individuals with no detectable levels of antibody in urine.

### Elevated levels of urine IgG to OV-antigen can detect individuals who are APF+ compared to EN or APF− individuals

Table 5 shows that elevated levels of urine IgG could discriminate between individuals who were APF positive from EN individuals, with an AUC of 0.72 and a PPV of 0.67 using a cutoff of 4.00 AUs of urine IgG to OV antigen. In addition, urine IgG to OV antigen showed significant crude and adjusted ORs for predicting risk of APF: crude OR = 6.34 (95%CI: 2.75, 14.66) and adjusted OR = 6.69 (95%CI: 2.87, 15.58). Elevated levels of urine IgG to OV antigen could also differentiate individuals with APF from matched APF− controls as well as an OR which indicated risk of APF in adjusted and unadjusted models. Table S2 shows that serum IgG to OV antigen could also modestly discriminate APF positive individuals from individuals from the EN group, with an AUC of 0.52 and a PPV of 0.52 and an adjusted OR of 2.71 (95%CI: 1.26, 5.84).

**Table 5:** Clinical epidemiology of serum and urine IgG to OV antigen to detect APF and CCA.

						Odds Ratio (95%CI Lower, Upper)	
Groups	AUC†	Cut Off (AU*)	Sensitivity (95%CI)	Specificity (95%CI)	PPV**	Crude	Adjusted^a^
APF+ vs. EN‡	**0.72**	>4.00	**0.67** (0.58, 0.75)	**0.66** (0.22, 0.96)	**0.67**	**6.34** (2.75, 14.66)	**6.69** (2.87, 15.58)
APF+ vs. APF−	**0.65**	>0.89	**0.71** (0.61, 0.79)	**0.52** (0.40, 0.63)	**0.60**	**1.20** (1.23, 3.26)	**1.98** (1.21, 3.23)
CCA vs. EN	**0.97**	>2.84	**1.00** (0.63, 1.00)	**0.73** (0.45, 0.92)	**0.79**	**85.00** (19.89, 362.0)	**71.13** (15.13, 334.0)

† Area Under the Curve;

* Arbitrary Units of antibody;

** Positive predictive value;

‡ EN refers to Endemic Normals (Group 1);

a Adjusted for age and sex.

APF refers to advanced perdicutal fibrosis as determined by abdominal ultrasound. CCA refers to confirmed cholangiocarcinoma. The positive predictive value (PPV) used a prevalence of 50% from field studies in [7], [8], [18]. Estimations of risk by Odds Ratios and 95% Confidence Intervals based on the “cut-offs” obtained by Receiver Operator Characteristic (ROC) curve analyses. Odds Ratios were adjusted for age and sex. Odds Ratios calculated against individuals with no detectable levels of antibody in urine.

### Levels of urine IgG to OV-antigen can discriminate between individuals with confirmed CCA against other OV-related pathologies

Table 5 also shows that elevated levels of urine IgG to OV antigen can discriminate better than serum antibodies individuals with confirmed CCA versus endemic normals, with an AUC of 0.97 for the ROC curve analysis and a PPV of 0.79 when the antibody cutoff of 2.84 AU is used. In addition, Table 5 shows that elevated levels of urine IgG to OV antigen are strongly associated with CCA: crude OR = 85.00 (95%CI: 19.89, 362.0) and adjusted for sex and age OR = 71.13 (95%CI: 15.13, 334.0). Table S3 shows that serum antibodies to OV antigen do not have the sensitivity nor specificity to be predictive of CCA nor the crude and adjusted Odds Ratios to indicate risk for CCA compared to urine IgG to OV antigen.

## Discussion

**Figure 3:**
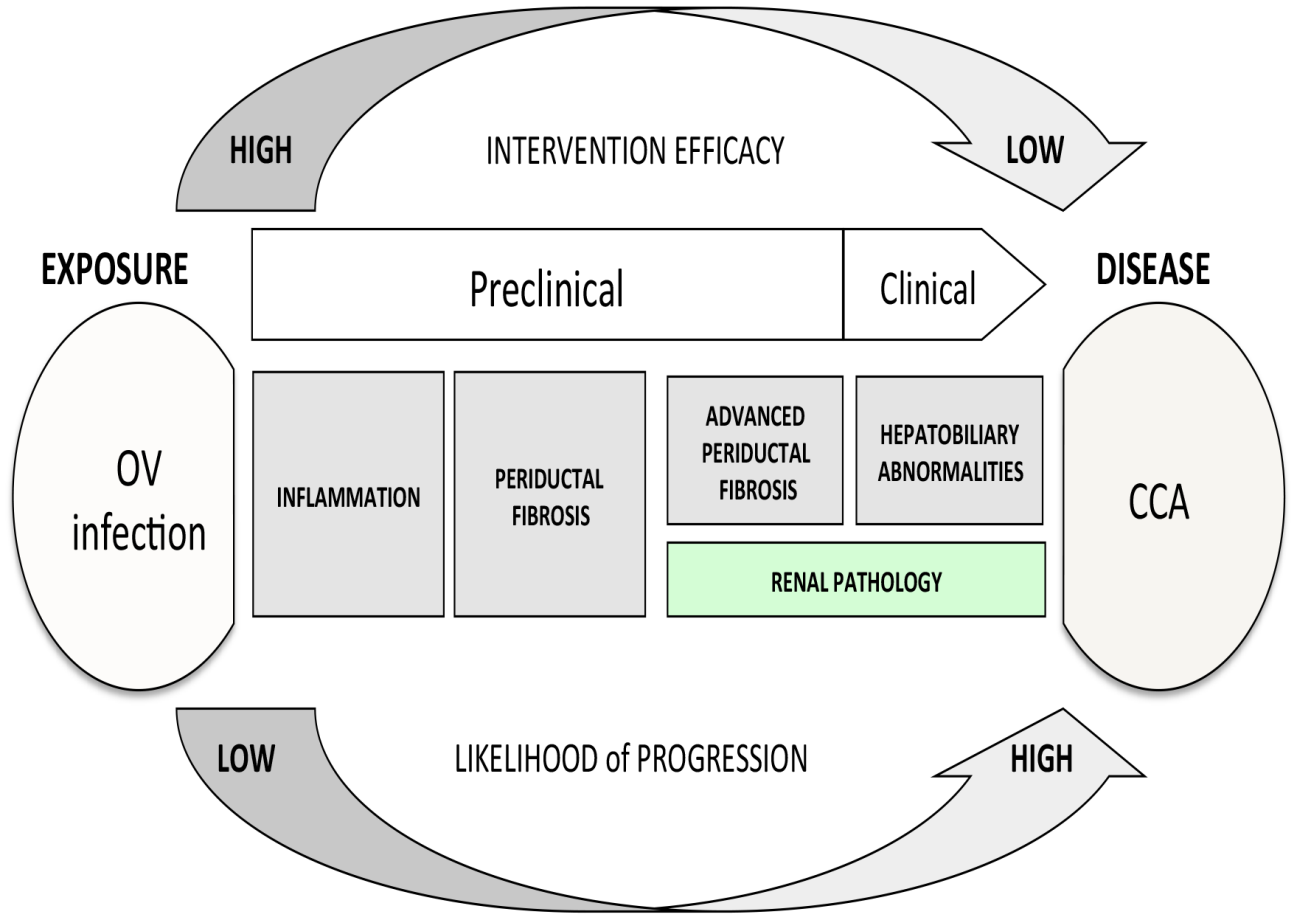
Renal pathology in the progression from *Opisthorchis viverrini* infection to cholangiocarcinoma. Figure 3 is an adaption of the pathway to pathogenesis of OV infection as previously published in [40]. Here, we have added in the green box the role of renal pathology the form of a microproteinuria in the progression from chronic opisthorchiasis to CCA. This renal pathology most likely results from sustained systemic effects of the parasitic infection on the host immune response (i.e., immune complex–mediated glomerulopathy). Despite the lack of a common pathogenic mechanism, the renal and hepatobiliary pathologies associated with OV infection develop simultaneously in the laboratory animal model and (as hypothesized in this manuscript) in humans chronically infected with OV as well. As such, a biomarker for renal pathology could be equally indicative of risk for APF and CCA, i.e., “syndromic biomarker” for the advanced pathologies associated with chronic opisthorchiasis.

In the current manuscript, we show that more than half of the individuals resident in endemic areas along the Chi River Basin in Khon Kaen, northeastern (Isaan) Thailand have detectable levels of urine IgG to adult OV antigen extract. Moreover, elevated levels of urine IgG to OV antigen were not associated with either the intensity of OV infection (as measured by fecal egg counts) or the levels of serum antibodies to OV antigen. These findings support our hypothesis that urine IgG to OV antigen most likely represents renal pathology in the form of immune complex-mediated structural damage to the glomeruli (injury of podocytes) and to the tubular interstitium as observed in animal models of OV infection [10], [11]. Interestingly, we found that IgG to OV antigen was detectable in the urine at concentrations 21-times higher in individuals with OV-induced APF and 158-times higher in individuals with OV induced CCA compared to controls. As shown in Table 5, individuals with elevated urine IgG to OV antigen had an increased risk for APF (adjusted OR of 6.69; 95%CI: 2.87, 15.58) and an increased risk for CCA (adjusted OR of 71.13; 95%CI: 15.13, 334.0) than individuals who had no detectable levels of urine IgG to OV antigen. We also found that a single measurement of urine IgG to OV antigen had good predictive value for the detection of both APF and CCA compared to age, sex, and nearest-neighbor matched non-cases (either EN or APF negative controls). Hence, as shown in Figure 3, our study adds to the literature on the pathophysiology of opisthorchiasis the possibility that renal pathology occurs simultaneously with the advanced hepatobiliary pathologies more commonly associated with this infection and that renal pathology can be detected by elevated levels of urine IgG to OV antigen [10], [11]. A urine-based assay that could simultaneously evaluate the clinical status of individuals for both renal and hepatobiliary pathologies from chronic opisthorchiasis would be of profound benefit in Southeast Asia, especially in the resource-limited settings of the Mekong Basin region countries of Thailand, Laos and Cambodia.

Antibodies have long been known to play a central role in the immune response to *Opisthorchis viverrini* infection [14], [26]–[36]. Individuals and animals infected with this food-borne trematode show high serum/plasma levels of the classic antibodies associated with helminth infections such as IgG, IgG1, IgG4 and IgE to crude OV antigen extracts. As such, it has been hypothesized that circulating antibodies to OV antigens may “leak” from the plasma into the urine at levels proportionate to the intensity of OV infection [14], [16]. However, as seen in these other studies [14], [16], we found that urine IgG to OV antigen is a poor method for diagnosing OV infection and an even poorer method for predicting the intensity of OV infection (Tables 3 and 4). In addition, we observed only weak correlations between circulating levels of serum IgG to OV antigen and levels of urine IgG to OV antigen. These findings are consistent with our current understanding of the pathophysiology of urine proteinuria (e.g. IgG in urine) from various clinical settings [9], [11], [17], [37], [38]. A healthy glomerular capillary wall should efficiently restrict the passage of IgG from the blood (plasma) into Bowman's space on the basis of this intact immunoglobulin's molecular size, electrical charge, and steric configuration; for example, the restrictive pore radius of the renal glomerular filter is 45 Ångstroms (Å), whereas intact IgG has a molecular radius of 55 Å (see [17] for excellent review). Additionally, IgG is a cationic protein, which means it binds strongly to the negatively charged proximal tubule cells [17]. Hence, even if small amounts of IgG are filtered into Bowman's space and, thereby into the tubular lumen, they would be readily reabsorbed in the proximal tubule. In keeping with similar findings in experimental animal models of OV infection [10], we hypothesize that the frequent observation of IgG in the urine of OV infected individuals reflects glomerulopathy. More specifically, we suspect that it reflects structural damage to the glomerular capillary wall characterized by injured podocytes, resulting in increased glomerular permeability or increased glomerular pore size that allows for passage of macromolecules such as IgG. In addition, the reabsorption capacity of the epithelial cells of the proximal tubules may also be impaired. Both glomerular and tubular damage are cardinal signs of kidney disease [17], [37], [38].

The fact that the IgG detected in the urine is specific for OV antigen leads us to postulate that the observed renal pathology is the result of immune complex deposition similar to that observed in animal models of OV [10]. Renal pathology caused by immune complex deposition has been described in association with other parasitic helminth infections such as schistosomiasis [9]. Immune complexes are putatively deposited in the glomerular subendothelium, resulting in activation of complement, chemoattraction of leukocytes, and an inflammatory reaction that leads to disruption of the glomerular basement membrane with enlargement of glomerular barrier pores, thus permitting passage of high molecular weight (HMW) macromolecules such as IgG to which the basement membrane is normally impermeable [17], [38]. The increased load of HMW macromolecules in the tubular lumen leads to saturation of the re-absorptive mechanism by tubular cells. As mentioned above, the renal pathology observed in OV-infected hamsters is a mesangial proliferative glomerulonephritis with the immune complexes consisting of IgG, complement component 3 (C3), and OV antigen [10], [11], [39]. Immune complexes accumulate in the glomeruli of the hamster kidney either by (a) passive trapping of circulating immune complexes or (b) *in situ* formation by the binding of antibody to OV-antigen that was previously deposited in the glomeruli. During the course of a single experimental OV infection, hamsters develop progressive sclerosis of glomeruli, tubular atrophy, as well as interstitial inflammation and fibrosis that appear to be coincident with the development of bile duct fibrosis and bile duct cancer [10].

However, not all individuals, who are chronically infected with *O. viverrini*, develop renal or hepatobiliary abnormalities. From our community-based studies in OV endemic areas in northeastern Thailand, we have observed that a subset of individuals infected with OV respond to the chronic inflammation from OV infection response with pathologies [7], [8], [18]. We have termed these individuals as having a ‘pro-inflammatory’ phenotype [40]. The current study adds to our hypothesis evidence that the ‘pro-inflammatory’ phenotype extends to renal pathology associated with chronic opisthorchiasis (i.e., APF and CCA). We suspect that individuals with the ‘pro-inflammatory phenotype’ have a dysregulation of inflammatory cytokine production in response to chronic fluke infection, which manifests as inflammation in the renal filters (glomeruli), enlarging them to allow passage of macroproteins (e.g., intact IgG to OV antigen) from the plasma. As seen in the hamster model, we also suspect that over time there is a progressive obsolescence of the glomeruli, tubular atrophy, interstitial inflammation, and renal fibrosis associated with this proteinuria [10], [11], [39]. As urine IgG to OV antigen is an easily accessible biomarker, we suggest that it could serve as a biomarker for the multiple inflammation-related pathologies from opisthorchiasis.

An important question arising from our study is the absence of detectable proteinuria by means of point-of-care testing using a strip-based urine reagent device. Theoretically, the increased permeability of the glomerular barrier that allows the filtering of IgG should also result in proportional losses of albumin into the urine, which is the principal protein component detected by the urine dipstick test [37]. There are two possibilities that explain the false negative urine dipstick results. First, total protein concentration in urine depends on degree of hydration (i.e., the specific gravity of the urine). False negative results may relate to the manner in which the urine samples were collected [37], including collection of the entire urine sample and not a mid-stream urine sample (which represents urine from the kidneys) or the collection of first morning urine (24 hour urine sample), which represents an accumulation of urine that could result in a dilution of the sample that would decrease the sensitivity of the dipstick test to detect proteinuria [37]. On the other hand, the indirect ELISA would be much more sensitive to the presence of IgG in urine than a dipstick test because (a) the urine is concentrated in a preparation step prior to immunoassaying and (b) the monoclonal antibodies to human IgG used in the ELISA are much more sensitive and specific than the dipstick's colorimetric method which is used to primarily detect albumin in the urine. The second hypothesis is the possibility of a restrictive mechanism that prohibits the filtering of albumin into urine but that allows leakage of IgG. Numerous investigators (see [37] for review) have observed that the movement of albumin into Bowman's space is not restricted by pore size, but by its negative charge and the consequent repulsive electrostatic interactions with the negatively charged glomerular endothelium. Hence, it is quite possible that the presence of IgG in the urine due to OV infection is not accompanied by appreciable albuminuria—a hypothesis that clearly deserves further study.

A final issue is the relationship between the concurrent renal and hepatobiliary pathologies observed in this study. The strongest associations in the current study were between elevated urine IgG to OV antigen and APF or CCA: individuals with elevated levels of urine IgG to OV antigen had a 6 times greater risk of having APF and a 71-times greater risk of having CCA (Table 5 adjusted OR) than individuals with no detectable IgG to OV antigen in their urine. Additionally, a single measurement of urine IgG to OV antigen had a good positive predictive value for the detection of both APF and CCA. However, as shown in the hamster model for OV-induced bile duct fibrosis and CCA [10], no plausible physiologic relationship exists between the hepatobiliary and renal pathologies induced by chronic OV infection. It appears that they are the result of two distinct pathological mechanisms that develop simultaneously during OV infection. As we have written extensively, hepatobiliary pathology from chronic opisthorchiasis is likely the result of repeated injury sustained by the biliary epithelium from a combination of the mechanical, toxic, and immune insults associated with the presence of the fluke in the bile duct [for review see [8], [18]). As individuals are infected with *O. viverrini* for many years (often a lifetime), a persistent cycle of tissue damage and repair takes place in the intrahepatic biliary ducts, creating a chronic inflammatory milieu that stimulates periductal fibrogenesis and tumorigenesis [8], [18]. The renal injury observed herein is the likely the consequence of chronic OV infection, resulting from the sustained systemic effects of the parasitic infection on the host immune response (i.e., immune complex–mediated glomerulopathy).

Despite the lack of a common pathogenic mechanism, the renal and hepatobiliary pathologies associated with OV infection develop simultaneously in the animal model and probably in humans as well. As such, a biomarker for renal pathology could be equally indicative of risk for APF and CCA. It should also be noted that as early as 1990, Mairiang et al. [12], reported acute renal failure in nearly all patients with obstructive jaundice due to CCA caused by opisthorchiasis. This is in keeping with our own findings of elevated urine IgG to OV antigen in CCA cases. However, in the case of end-stage CCA, the finding of proteinuria may reflect “hepatorenal syndrome” (HRS), which is a common complication of patients with advanced forms of liver disease such as CCA and cirrhosis [13] and is caused by intense vasoconstriction of the renal circulation, leading to a pronounced reduction in glomerular perfusion and filtration [13]. HRS generally occurs in late stages of severe liver disease, when patients have already manifested significant complications of cirrhosis. HRS is an acute condition with a very poor prognosis. As such, there remains some question as to whether the renal pathology, presumably chronic in nature, that is seen among individuals with opisthorchiasis-induced APF is the same as that seen among CCA cases, as the latter may reflect HRS rather than immune complex associated glomerulonephritis.

As shown by our community-based studies [7], [8], [18], chronic *O. viverrini* infection results in a persistent immunological and inflammatory challenge to the human host. For the first time, we have shown that chronic OV infection may also result in a significant burden of renal disease in the form of immune complex-mediated glomerulopathy. The importance of this study is the observation that this renal pathology can be readily detected in the urine by an immunoassay for IgG against OV-antigen and that elevated levels of urinary IgG to OV-antigen are also strongly associated with hepatobiliary pathologies. In future studies, we plan to improve on the sensitivity and specificity of this biomarker by screening urine for the specific antigens recognized by IgG in the crude adult OV-antigen extract used here. Recent advances in immunomics, in which the *O. viverrini* proteome can be assembled on a microarray chip, allows for high-throughput screening of urine samples to determine the most abundantly recognized proteins. These could subsequently be developed as recombinant proteins as reagents for urine diagnostic tests. As such, screening for urinary IgG to specific recombinant OV antigens might be used to indicate risk of several pathologies that can arise from chronic opisthorchiasis, and thereby be used as a “*syndromic biomarker*” of chronic opisthorchiasis.

## Supporting Information

Checklist S1 **STROBE Checklist.**
(DOC)

Figure S1 **Performance characteristics for ELISAs to detect antibodies in serum and urine to** ***O. viverrini*** **antigen.** Panel A shows the mean and 95% CI for 12 Standard Calibration Curves (SCCs) for serum IgG to OV antigen and Panel B shows the estimation of the RDL. Panel C shows the mean and 95% CI for 12 SCC for serum IgG1 to OV-antigen and Panel D shows the estimation of the RDL. Panel E shows the mean and 95% CI for 12 SCC for serum IgG4 to OV antigen and Panel F shows the estimation of the RDL. Panel G shows the mean and 95% CI for 10 SCCs for urine IgG to OV antigen and Panel H shows the estimation of the RDL.
(PDF)

Figure S2 **Parallelism for Standard Calibration Curves to detect IgG to OV antigen in serum and urine.** The linearized 4 parameter logistic log (4-PL) modeling of either a Standard Reference Serum (for IgG, IgG1, and IgG4) or a urine Standard Reference Solution for IgG to OV antigen. Each SRS is serially diluted on an ELISA plate where the Optical Density (OD) 492 nm is plotted against log_10_ of the dilution. The horizontal axis in each panel represents the log dilution of each SRS and the vertical axis represents the *logit* of the Optical Density (OD) at 492 nm. The sigmoidal 4PL lines are linearized and compared by for parallelism. Panel A shows an analysis of parallelism of the SCCs for serum IgG to OV antigen; Panel B for serum IgG1 to OV antigen; Panel C to serum IgG4 against OV antigen; and Panel D urine IgG against OV antigen. A p≥0.05 shows a non-significant departure from parallelism.
(PDF)

Figure S3 **Levels of serum IgG and urine IgG to OV antigen and proteinuria.** Panel A shows the linear relationship between Arbitrary Units of serum IgG and urinary IgG to a crude OV antigen extract in the 256 individuals who are OV positive in the study. Panel B shows the levels of proteinuria by clinical groups as determine by point-of care urine dipstick.
(PDF)

Table S1 **Serum and urine IgG to OV antigen for the detection of APF versus Endemic Normal individuals.**
(DOCX)

Table S2 **Serum antibodies to OV antigen for the detection of cholangiocarcinoma cases compared to endemic normals.**
(DOCX)

Table S3 **Improved diagnostic capability using homologous interpolation and Arbitrary Units for the indirect ELISA.**
(DOCX)
