# Correction: Microproteinuria during *Opisthorchis viverrini* Infection: A Biomarker for Advanced Renal and Hepatobiliary Pathologies from Chronic Opisthorchiasis

**DOI:** 10.1371/annotation/22389ccb-213a-4502-a8f5-e1993d885cfa

**Published:** 2013-08-23

**Authors:** Prasert Saichua, Paiboon Sithithaworn, Amar R. Jariwala, David J. Diemert, Jiraporn Sithithaworn, Banchob Sripa, Thewarach Laha, Eimorn Mairiang, Chawalit Pairojkul, Maria Victoria Periago, Narong Khuntikeo, Jason Mulvenna, Jeffrey M. Bethony

The fourth author's name was misspelled in the author list and citation. The correct name is David J. Diemert.

The citation should read: Saichua P, Sithithaworn P, Jariwala AR, Diemert DJ, Sithithaworn J, et al. (2013) Microproteinuria during Opisthorchis viverrini Infection: A Biomarker for Advanced Renal and Hepatobiliary Pathologies from Chronic Opisthorchiasis. PLoS Negl Trop Dis 7(5): e2228. doi:10.1371/journal.pntd.0002228 

