# Supplementary figures and images for: Microproteinuria during Opisthorchis viverrini Infection: A Biomarker for Advanced Renal and Hepatobiliary Pathologies from Chronic Opisthorchiasis
Source: PLoS Negl Trop Dis. 2013 May 23;7(5):e2228. doi: 10.1371/journal.pntd.0002228 (PMC3662652; doi:10.1371/journal.pntd.0002228)

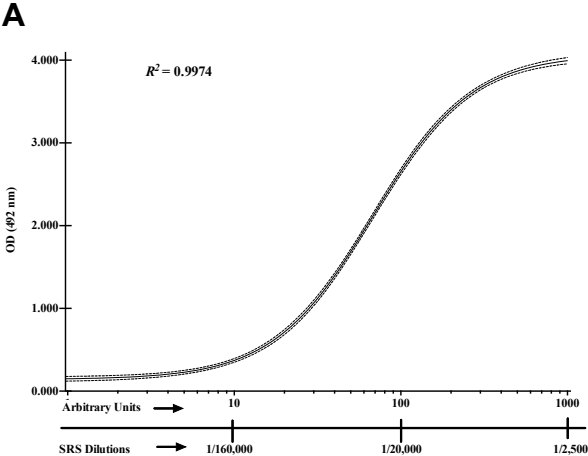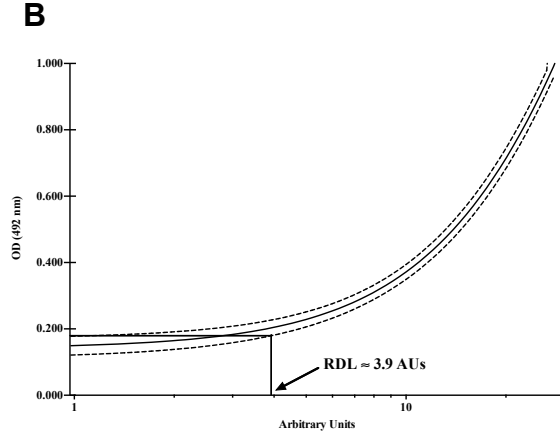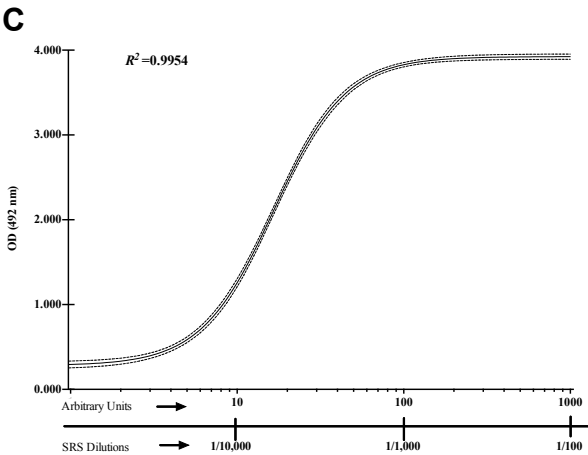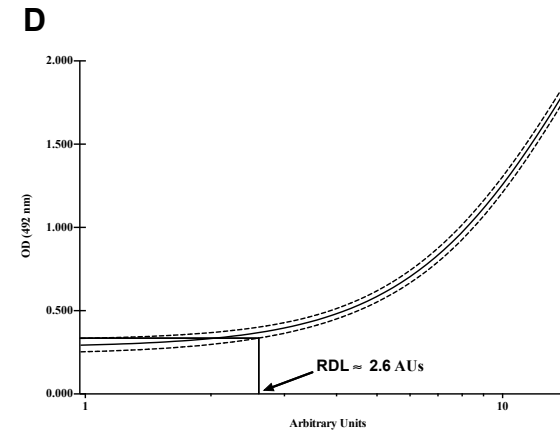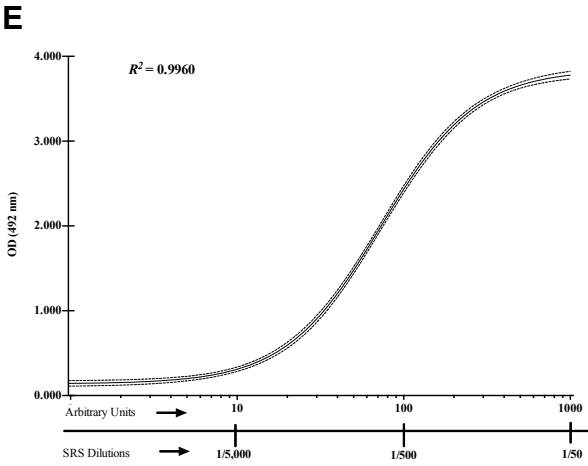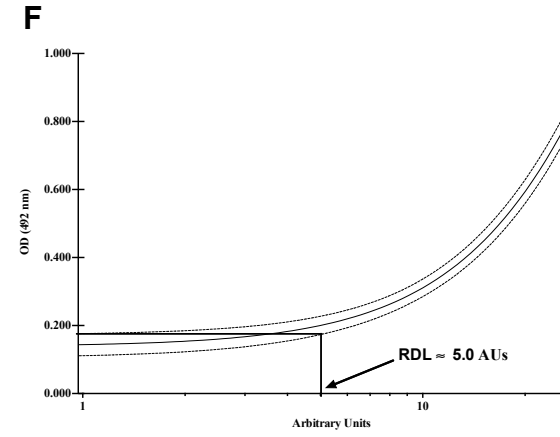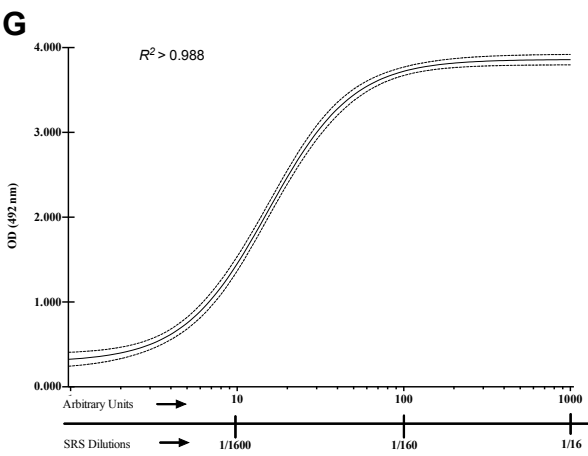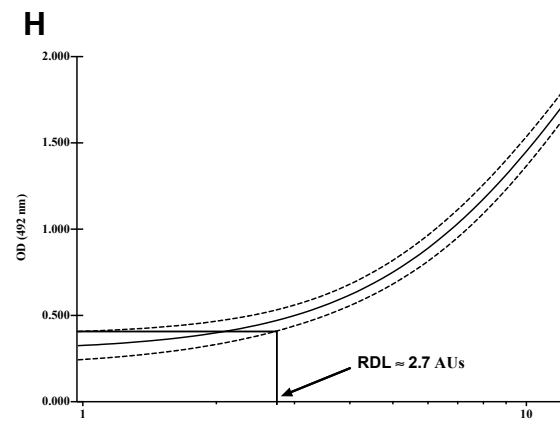

Supplement: Figure S1 — Performance characteristics for ELISAs to detect antibodies in serum and urine to O. viverrini antigen. Panel A shows the mean and 95% CI for 12 Standard Calibration Curves (SCCs) for serum IgG to OV antigen and Panel B shows the estimation of the RDL. Panel C shows the mean and 95% CI for 12 SCC for serum IgG1 to OV-antigen and Panel D shows the estimation of the RDL. Panel E shows the mean and 95% CI for 12 SCC for serum IgG4 to OV antigen and Panel F shows the estimation of the RDL. Panel G shows the mean and 95% CI for 10 SCCs for urine IgG to OV antigen and Panel H shows the estimation of the RDL. (PDF) [file pntd.0002228.s002.pdf]

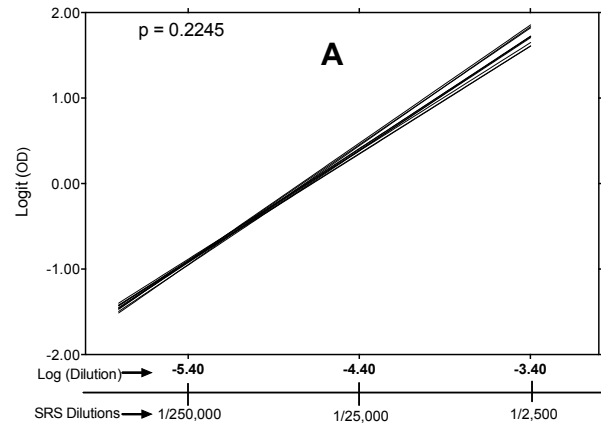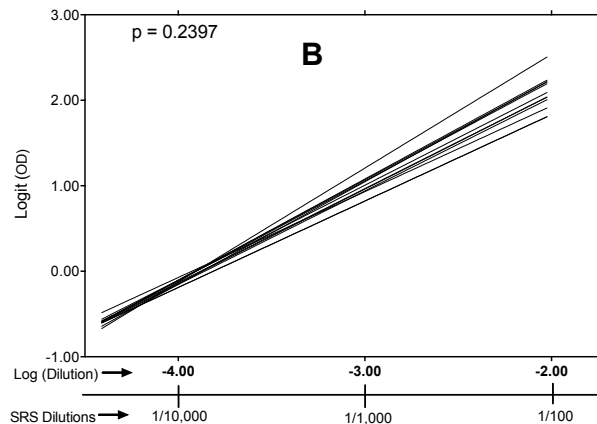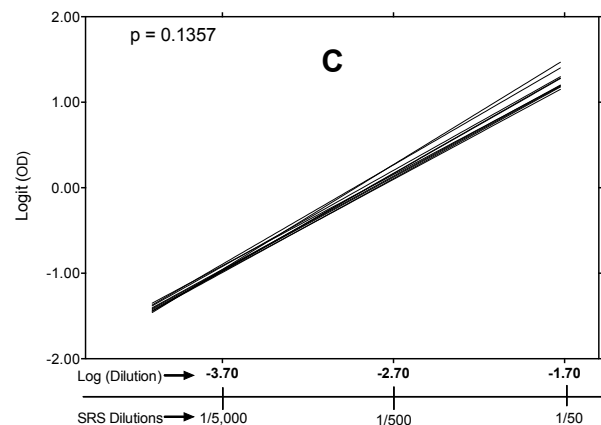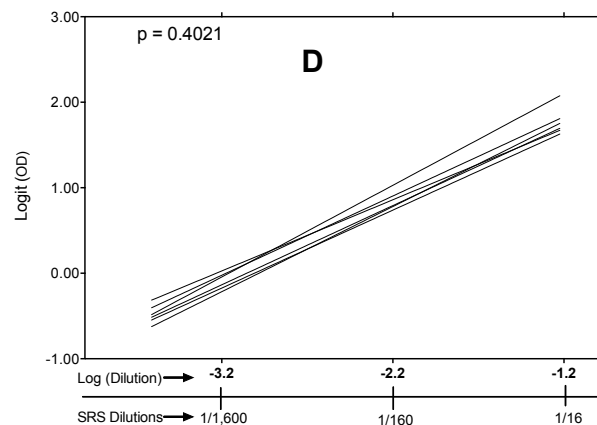

Supplement: Figure S2 — Parallelism for Standard Calibration Curves to detect IgG to OV antigen in serum and urine. The linearized 4 parameter logistic log (4-PL) modeling of either a Standard Reference Serum (for IgG, IgG1, and IgG4) or a urine Standard Reference Solution for IgG to OV antigen. Each SRS is serially diluted on an ELISA plate where the Optical Density (OD) 492 nm is plotted against log10 of the dilution. The horizontal axis in each panel represents the log dilution of each SRS and the vertical axis represents the logit of the Optical Density (OD) at 492 nm. The sigmoidal 4PL lines are linearized and compared by for parallelism. Panel A shows an analysis of parallelism of the SCCs for serum IgG to OV antigen; Panel B for serum IgG1 to OV antigen; Panel C to serum IgG4 against OV antigen; and Panel D urine IgG against OV antigen. A p≥0.05 shows a non-significant departure from parallelism. (PDF) [file pntd.0002228.s003.pdf]
