# Supplementary material for: Microproteinuria during Opisthorchis viverrini Infection: A Biomarker for Advanced Renal and Hepatobiliary Pathologies from Chronic Opisthorchiasis
Source: PLoS Negl Trop Dis. 2013 May 23;7(5):e2228. doi: 10.1371/journal.pntd.0002228 (PMC3662652; doi:10.1371/journal.pntd.0002228)
Supplement: Table S1 — Serum and urine IgG to OV antigen for the detection of APF versus Endemic Normal individuals. (DOCX) [file pntd.0002228.s005.docx]

**Table S1.** Improved diagnostic capability using homologous interpolation and Arbitrary Units for the indirect ELISA.

| Dilution | AU |
| --- | --- |
| 1:400 | 1000.00 |
| 1:800 | 500.00 |
| 1:1,600 | 250.00 |
| 1:3,200 | 125.00 |
| 1:6,400 | 62.50 |
| 1:12,800 | 31.25 |
| 1:25,600 | 15.63 |
| 1:51,200 | 7.81 |
| 1:102,400 | 3.91 |
| 1:204,800 | 1.95 |
| 1:409,600 | 0.98 |
| 1:819,200 | 0.49 |

Homologous interpolation to derive Arbitrary Units (AU) of IgG to OV antigen from a Standard Calibration Curve derived from either a Standard Reference Serum of IgG against OV antigen or a Standard Reference Solution of urine IgG against OV antigen. The two-fold serial dilutions of the Standard Reference Serum or Standard Reference Solution of urine started at a dilution of 1:400 and were assigned 1,000 Arbitrary Units, a number chosen for convenience when making calculations. The subsequent two-fold dilutions (e.g. 1:800, 1:1,600, etc.) were assigned corresponding AUs by serially dividing the 1000 by two (2). The AUs of the Standard Reference Serum and Standard Reference Solution were obtained by interpolating its ODs (492nm) onto the 4-PL Standard Calibration Curve and then finding the corresponding values on the vertical axis, which are expressed as AUs. This interpolation of the AUs was done using the SoftMax Pro GxP 5.4.1 software.
