# Supplementary material for: Microproteinuria during Opisthorchis viverrini Infection: A Biomarker for Advanced Renal and Hepatobiliary Pathologies from Chronic Opisthorchiasis
Source: PLoS Negl Trop Dis. 2013 May 23;7(5):e2228. doi: 10.1371/journal.pntd.0002228 (PMC3662652; doi:10.1371/journal.pntd.0002228)
Supplement: Table S2 — Serum antibodies to OV antigen for the detection of cholangiocarcinoma cases compared to endemic normals. (DOCX) [file pntd.0002228.s006.docx]

| **Table S2.** Serum IgG to OV antigen for the detection of APF versus Endemic Normal individuals. | | | | | | | | |
| --- | --- | --- | --- | --- | --- | --- | --- | --- |
|  |  |  |  |  |  | Odds Ratios (95%CI Lower, Upper) | |  |
| Assay | AUC^†^ | Cut Off (AUs*) | Sensitivity (95%CI) | Specificity (95%CI) | PPV^**^ | Crude | Adjusted^a^ |  |
| Serum IgG | 0.52 | > 35.92 | 0.80 (0.75, 0.85) | 0.50 (0.34, 0.66) | 0.52 | 2.79 (1.30, 5.98) | 2.71 (1.26, 5.84) |  |
| Serum IgG1 | 0.51 | > 9.29 | 0.80 (0.75, 0.85) | 0.51 (0.35, 0.68) | 0.62 | 2.67 (1.25, 5.73) | 2.50 (1.16, 5.37) |  |
| Serum IgG4 | 0.50 | > 16.66 | 0.66 (0.54, 0.76) | 0.63 (0.24, 0.91) | 0.64 | 2.54 (1.13, 5.72) | 2.32 (1.02, 5.24) |  |
| **^†^**Area Under the Curve; *Arbitrary Units of antibody; **^**^**Positive predictive value; **^a^**Adjusted for age and sex. APF refers to advanced perdicutal fibrosis as determined by abdominal ultrasound. The positive predictive value (PPV) used a prevalence of 50% from field studies in [[7](#_ENREF_7), [8](#_ENREF_8), [18](#_ENREF_18)]. Estimations of risk by Odds Ratios and 95% Confidence Intervals were based on the “cut-offs” obtained by Receiver Operator Characteristic (ROC) curve analyses. Odds Ratios were adjusted for age and sex. | | | | | | | | |
