# Supplementary material for: Microproteinuria during Opisthorchis viverrini Infection: A Biomarker for Advanced Renal and Hepatobiliary Pathologies from Chronic Opisthorchiasis
Source: PLoS Negl Trop Dis. 2013 May 23;7(5):e2228. doi: 10.1371/journal.pntd.0002228 (PMC3662652; doi:10.1371/journal.pntd.0002228)
Supplement: Table S3 — Improved diagnostic capability using homologous interpolation and Arbitrary Units for the indirect ELISA. (DOCX) [file pntd.0002228.s007.docx]

| **Table S3**. Serum antibodies to OV antigen for the detection of cholangiocarcinoma cases compared to endemic normals. | | | | | | | |
| --- | --- | --- | --- | --- | --- | --- | --- |
|  | | | | | |  | |
|  |  |  |  |  |  | Odds Ratio (95%CI Lower, Upper) | |
| ELISA | AUC^†^ | Cut Off (AUs*) | Sensitivity (95%CI) | Specificity (95%CI) | PPV^**^ | Crude | Adjusted^a^ |
| IgG | 0.57 | > 35.26 | 0.54 (0.44, 0.64) | 0.50 (0.34, 0.66) | 0.52 | 1.16 (0.51, 2.63) | 0.73 (0.27, 1.94) |
| IgG1 | 0.57 | > 8.45 | 0.62 (0.512, 0.72) | 0.49 (0.32, 0.65) | 0.55 | 1.39 (0.61, 3.14) | 0.99 (0.37, 2.62) |
| IgG4 | 0.40 | > 8.29 | 0.63 (0.53, 0.73) | 0.44 (0.28, 0.60) | 0.43 | 0.47 (0.20, 1.09) | 0.56 (0.20, 1.56) |
| **^†^**Area Under the Curve; Arbitrary Units of antibody; **^**^**Positive predictive value; **^a^**Adjusted for age and sex. The positive predictive value was ascertained by using 50% prevalence from field studies in [[7](#_ENREF_7), [8](#_ENREF_8), [18](#_ENREF_18)]. Estimations of risk by Odds Ratios and 95% Confidence Intervals were based on the “cut-offs” obtained by Receiver Operator Characteristic (ROC) curve analyses. Odds Ratios were adjusted for age and sex. Odds Ratios calculated against individuals with no detectable levels of antibody in urine. | | | | | | | |
