# Supplementary material for: Microproteinuria during Opisthorchis viverrini Infection: A Biomarker for Advanced Renal and Hepatobiliary Pathologies from Chronic Opisthorchiasis
Source: PLoS Negl Trop Dis. 2013 May 23;7(5):e2228. doi: 10.1371/journal.pntd.0002228 (PMC3662652; doi:10.1371/journal.pntd.0002228)
Supplement: Figure S3 — Levels of serum IgG and urine IgG to OV antigen and proteinuria. Panel A shows the linear relationship between Arbitrary Units of serum IgG and urinary IgG to a crude OV antigen extract in the 256 individuals who are OV positive in the study. Panel B shows the levels of proteinuria by clinical groups as determine by point-of care urine dipstick. (PDF) [file pntd.0002228.s004.pdf]

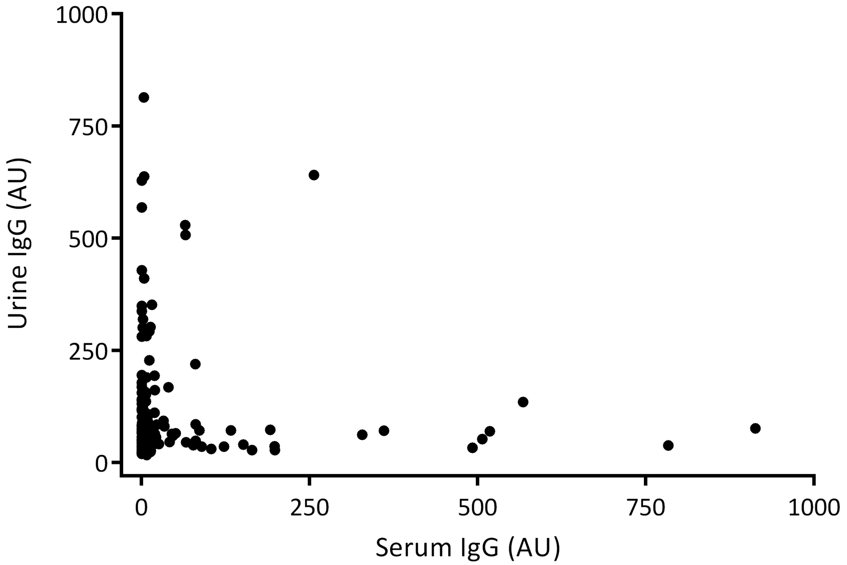

Urine IgG (AU)
1000
750
500
250
0
0
250
500
750
1000
Serum IgG (AU)

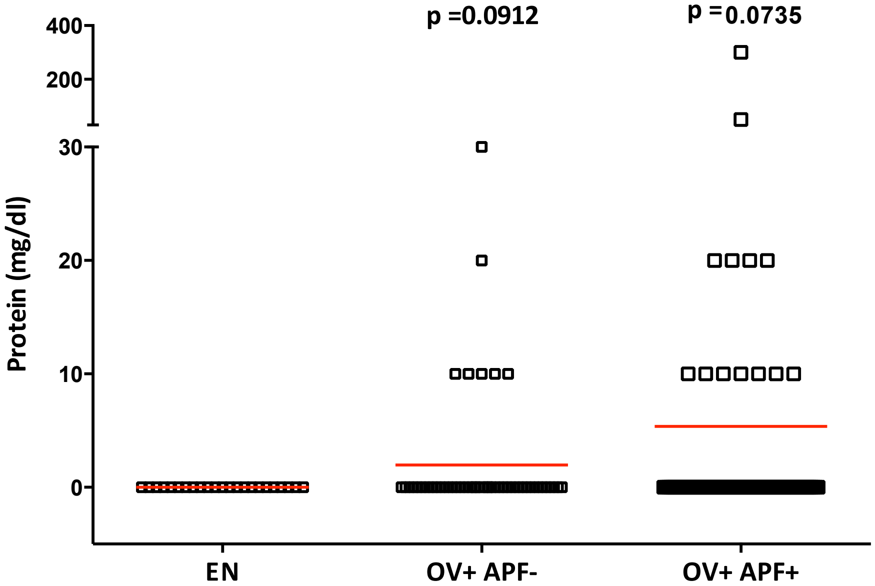

p =0.0912
p =0.0735
400
200
30
20
10
0
Protein (mg/dl)
EN
OV+ APF-
OV+ APF+
